# Global burden of soil-transmitted helminth infections, 1990–2021

**DOI:** 10.1186/s40249-024-01238-9

**Published:** 2024-10-24

**Authors:** Jin Chen, Yanfeng Gong, Qin Chen, Shizhu Li, Yibiao Zhou

**Affiliations:** 1 Fudan University School of Public Health, Fudan University Center for Tropical Disease Research, Shanghai, 200032 China; 2 National Institute of Parasitic Diseases, Chinese Center for Disease Control and Prevention; Chinese Center for Tropical Diseases Research; National Key Laboratory of Intelligent Tracking and Forecasting for Infectious Diseases; Key Laboratory on Parasite and Vector Biology, Ministry of Health; WHO Collaborating Centre for Tropical Diseases; National Center for International Research on Tropical Diseases, Ministry of Science and Technology, Shanghai, 200025 China; 3 School of Global Health, Chinese Center for Tropical Diseases Research, Shanghai Jiao Tong University School of Medicine, Shanghai, 200025 China

**Keywords:** Soil-transmitted helminth infections, Disease burden, Age, Gender, Geographical distribution, Socio-demographic index, Elimination

## Abstract

**Background:**

Soil-transmitted helminth (STH) infections can cause a significant disease burden. It is estimated that 1.5 billion people worldwide are infected with STHs, primarily in tropical and subtropical regions. This study aimed to assess the distribution of the global burden and trend of STH infections from 1990 to 2021.

**Methods:**

We retrieved data from the Global Burden of Diseases, Injuries, and Risk Factors Study 2021 on the age-standardized rates (ASRs) of prevalence and disability-adjusted life-years (DALYs) of STH infections for all age groups in 204 countries and territories from 1990 to 2021. The ASRs of prevalence and DALYs by age, gender, and socio-demographic index (SDI) were calculated to quantify the spatial distribution and temporal trend. Spearman correlation analysis was used to examine the relationship between ASR and SDI.

**Results:**

In 2021, there were an estimated 642.72 million cases and 1.38 million DALYs caused by STH infections worldwide. The age-standardized prevalence rate (ASPR) of STH infections was 8429.89 [95% uncertainty interval (UI): 7697.23, 9362.18 ] per 100,000 population globally. The ASPR of STH infections varied across 21 geographic regions in 2021, being mainly prevalent in most African and Latin American locations. The prevalence was higher in the groups of 5–19 years, especially the group of 5–9 years with the ASPR of 16,263 (95% UI: 14,877.06, 18,003.49) and ASR of DALYs of 40.69 (95% UI: 25.98, 60.91) per 100,000. The ASPR of STH infections showed an obvious decreasing trend worldwide (estimated annual percent change = − 4.03, 95% confidence interval: − 4.13, − 3.93) with variations in different species infections during the study years. At the regional level, the ASR of STH infections prevalence (*r* = − 0.8807, *P* < 0.0001) and DALYs (*r* = − 0.9069, *P* < 0.0001) were negatively correlated with SDI .

**Conclusions:**

STH infections remain a public health problem in 2021, particularly in regions such as the middle parts of Africa and Americas, and in the 5–19 age populations. The reduction in the rate of prevalence and the loss of DALYs are negatively correlated with the increase in SDI. Enhancing political commitment, providing accurate preventive chemotherapy, and advancing techniques for surveillance and mapping systems are essential to achieve the target of ending STH infections as a public health problem by 2030.

**Graphical Abstract:**

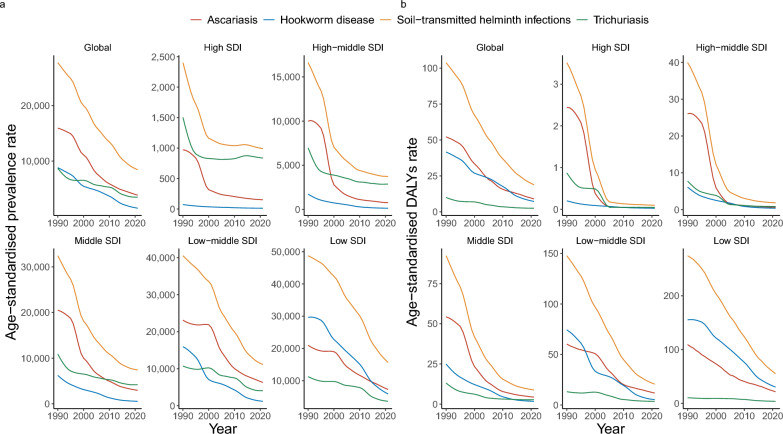

**Supplementary Information:**

The online version contains supplementary material available at 10.1186/s40249-024-01238-9.

## Background

Infectious diseases of poverty refer to a group of diseases that primarily affect the poorest populations, particularly those living in low-income regions of the world. These diseases have been divided into six catalogues based on the nature of pathogens [1]. Among them, soil-transmitted helminth (STH) infections mainly include infections with roundworm (*Ascaris lumbricoides*), whipworm (*Trichuris trichiura*), and hookworm infection. STH infections can cause a significant disease burden [2]. Although early and low-grade STH infections may cause mild symptoms, persistent infections can lead to severe complications and even death. Larvae of *Ascaris* can migrate within the body, leading to conditions such as larva migrants’ pneumonia and allergic symptoms. Adult worms reside in the small intestine, causing gastrointestinal dysfunction and mechanical bowel obstruction. Hookworm larvae migration can cause dermatitis and respiratory symptoms, while some adult hookworms can cause digestive and absorption disorders, particularly anemia. Adult whipworms inhabit the human intestine, which can cause mechanical damage to the intestinal wall tissue, leading to malnutrition, emaciation, fatigue, and iron deficiency anemia [3].

It is estimated that 1.5 billion people worldwide are infected with STHs, primarily in tropical and subtropical regions, while sub-Saharan Africa, South America, and South Asia have high prevalence rates [4]. The natural and soil conditions in these regions are conducive to the survival of STHs in the external environment. Lack of access to clean water, poor environmental and personal hygiene, and inadequate sanitation facilities further promote the spread of STH infections [5]. According to the assessment by World Health Organization (WHO), in 2020, over 914 million children and 138.8 million pregnant and lactating women worldwide lived in areas endemic for STH infections and needed preventive interventions or treatment.

Globally, WHO used Global Burden of Disease, Injuries, and Risk Factors Study (GBD) 2019 data to evaluate whether preventive chemotherapy interventions were associated with the decline in STH prevalence, and found a positive correlation in most regions and populations between 2000 and 2019 [6]. Another study by WHO assessed the global progress of STH control by 2020 using internal reporting data from control programs [4]. Some other studies estimated the number infected, risk factors, spatial and temporal distribution of STH infections, and identified the high-risk regions in South Asia, Africa using geostatistical analysis methods based on data extracted from literature [7–10]. GBD 2021 data have been used to analyze and/or project the global, regional and national burden for overall 371 diseases, 288 causes, 88 risk factors, and some specific diseases. This study aimed to analyze the distribution of STH infections in geographical regions and populations, the decline of prevalence during 1990–2021, and the association with socio-demographic index based on data of GBD 2021.

## Methods

### Study population and data collection

We obtained data on the age-standardized rate (ASR) of prevalence and disability-adjusted life-years (DALYs) of STH infections including ascariasis, hookworm diseases, and trichuriasis from GBD 2021 database using Global Health Data Exchange (GHDx). GBD 2021 provided estimates of the global burden of 371 diseases and injuries in 204 countries and territories over 1990 to 2021 [11]. We defined the study population as both genders and all age groups which were divided into 20 GBD age groups at 5-year intervals. The socio-demographic index (SDI) was calculated in GBD 2021 to represent the combined level of health-related social and economic conditions in each region. The SDI was the geometric mean of 0–1 indices of the total fertility rate in females younger than 25 years, mean education (years of schooling) for people aged 15 years and older, and the country’s lag-distributed income per capita. The 204 countries and territories in GBD 2021 were grouped into quintiles (low, low-middle, middle, high-middle, and high) based on country-level estimates of SDI in 2021 [12]. The 204 countries and territories in GBD 2021 were also grouped into 21 regions those are geographically close, epidemiologically similar, and share similar distributions of causes of death [11].

### Statistical analysis

The estimated annual percentage change in ASR was calculated to evaluate the average changing trends over the study time interval. The natural logarithm of ASR was assumed to fit the linear regression model $y=\alpha +\beta x+\varepsilon$, where $y$ is equal to ln(ASR), and $x$ refers to the calendar year. Then, the estimated annual rate change was equal to $100\times ({e}^{\beta }-1)$. 95% uncertainty intervals (UIs) were defined as the 25th and 975th values of the ordered 1000 draws. The 95% confidence interval (*CI*) of estimated annual percentage change were estimated using the linear regression model. An ASR was determined to represent an increasing or decreasing trend over time if both the estimated annual percentage change and its 95% *CI* were above or below 0, respectively. When the 95% *CI* included 0, the change in ASR was considered statistically non-significant [6, 11–13].

We used smoothing spline models to evaluate the relationship between the burdens of STH infections among all age and gender groups and SDI for the 21 regions and 204 countries and territories. The expected values were determined through a calculation that takes into account the SDI and disease rates across all locations. We fitted smooth splines using the Locally Weighted Scatterplot Smoothing method, which automatically determines the degree, number, and location of nodes (knots) based on the data and the span parameter [12]. Spearman correlation analysis was used to estimate the *r* indices and *P* values for the association of ASR with SDI [12]. *P* < 0.05 was considered statistically significant. All data analysis and mapping were processed with R software (v4.1.3, Lucent Technologies, Jasmine Mountain, USA).

## Results

### Overview of global burden of STH infections

#### Global burden of STH infections

In 2021, there were an estimated 642.72 million cases and 1.38 million DALYs caused by STH infections, among which, 293.80 million cases and 647.53 thousand DALYs caused by ascariasis, 112.82 million cases and 540.20 thousand DALYs caused by hookworm diseases, and 266.87 million cases and 193.92 thousand DALYs caused by trichuriasis. A total of estimated 3472 deaths caused by STH infections, mainly by ascariasis (Supplementary Table 1).

The global age-standardized prevalence rate (ASPR) of STH infections was 8429.89 (95% UI: 7697.23, 9362.18] per 100,000 population, decreased by 69.6% (95% UI: 0,65, 0.73) compared to that in 1990. Specifically, the ASPR of ascariasis was 3856.33 (95% UI: 3133.93, 4760.38) per 100,000, dropped by 75.8% (95% UI: 0.68, 0.81) compared with that in 1990. The global ASR of hookworm disease was 1505.49 (95% UI: 1418.67, 1598.89) per 100,000, fell by 82.9% (95% UI: 0.81, 0.85) compared with that in 1990. The ASPR of trichuriasis was 3482.27 (95% UI: 3147.21, 3898.86) per 100,000, reduced by 59.9% (95% UI: 0.51, 0.67) compared with that in 1990. When calculated by disability-adjusted life years (DALYs), the global ASR of DLAYs caused by STH infections was 18.84 (95% UI: 12.61, 27.31) per 100,000 in 2021, dropped by near 82% (95% UI: 0.80, 0.84) compared with that in 1990. While the global ASR of DLAYs per 100,000 caused by ascariasis, hookworm disease, and trichuriasis was 9.05 (95% UI: 6.16, 12.45), 7.26 (95% UI: 4.52, 11.11), and 2.53 (95% UI: 1.40, 4.13), respectively, with drop rate ranging from 74.7 to 82.7% (Supplementary Table 1).

#### Distribution

The prevalence of STH infections varied across 21 geographic regions in 2021 which were most prevalent in the majority of African and some regions of Latin American locations. The ASPR in Andean Latin America reached 30,153.70 (95% UI: 26,076.80, 34,143.48) per 100,000, followed by Central sub-Saharan Africa 22,494.62 (95% UI: 19,311.10, 25,719.52), while there were no/seldom cases estimated in Europe and Australasia. Similarly, Oceania with ASR of DALYs of 93.36 (95% UI: 57.09, 143.72) and Central sub-Saharan Africa with 65.50 (95% UI: 43.11, 98.66) per 100,000 suffered more from STH infections that any other regions (Table 1).

**Table 1:** ASR of prevalence and DALYs of STH infections in 21 geographic regions

Location	Prevalence (ASR per 100,000), 95% UI			DALYs (ASR per 100,000), 95% UI		
	1990	2021	Change	1990	2021	Change
Global	27,728.35 (25,660.30, 29,899.61)	8429.89 (7697.20, 9362.18)	− 0.70 (− 0.73, − 0.65)	103.83 (70.38, 153.46)	18.84 (12.61, 27.31)	− 0.82 (− 0.84, − 0.80)
High-income Asia Pacific	3572.76 (2444.42, 4982.81)	1834.10 (1122.75, 2796.61)	− 0.49 (− 0.71, 0.13)	0.10 (0.05, 0.16)	0.02 (0.01, 0.03)	− 0.85 (− 0.93, 0.68)
High-income North America	0.00 (0.00, 0.00)	0.00 (0.00, 0.00)	0.00 (0.00, 0.00)	0.00 (0.00, 0.00)	0.00 (0.00, 0.00)	NA
East Asia	33,688.89 (26,510.50, 41,752.12)	5024.45 (3374.11, 7233.87)	− 0.85 (− 0.91, − 0.77)	92.71 (50.09, 149.24)	1.76 (0.92, 2.91)	− 0.98 (− 0.99, 0.97)
South Asia	45,692.71 (39,898.06, 51,284.32)	10,033.41 (7313.85, 13,663.08)	− 0.78 (− 0.84, − 0.70)	150.02 (97.50, 225.76)	16.50 (9.51, 27.41)	− 0.89 (− 0.92, − 0.85)
Southeast Asia	45,079.31 (41,292.45, 48,220.89)	7444.31 (6249.87, 8931.38)	− 0.84 (− 0.86, − 0.80)	175.72 (113.86, 270.48)	14.68 (9.27, 22.66)	− 0.916 (− 0.93, − 0.90)
Central Asia	8266.248 (7047.34, 9838.85)	3574.27 (2931.33, 4498.00)	− 0.57 (− 0.67, − 0.44)	4.86 (2.97, 7.73)	2.13 (1.31, 3.34)	− 0.561 (− 0.68, − 0.40)
Eastern Europe	0.00 (0.00, 0.00)	0.00 (0.00, 0.00)	0.00 (0.00, 0.00)	0.00 (0.00, 0.00)	0.00 (0.00, 0.00)	NA
Central Europe	0.00 (0.00, 0.00)	0.00 (0.00, 0.00)	0.00 (0.00, 0.00)	0.00 (0.00, 0.00)	0.00 (0.00, 0.00)	NA
Western Europe	0.00 (0.00, 0.000)	0.00 (0.00, 0.00)	0.00 (0.00, 0.00)	0.00 (0.00, 0.00)	0.00 (0.00, 0.00)	NA
Caribbean	29,152.18 (26,058.55, 32,164.55)	10,407.60 (8906.03, 12,226.36)	− 0.64 (− 0.71, − 0.57)	87.21 (57.31, 132.09)	17.56 (11.11, 27.37)	− 0.80 (− 0.85, − 0.74)
North Africa and Middle East	7883.91 (6955.44, 8791.13)	6696.70 (5925.24, 7471.26)	− 0.15 (− 0.29, 0.01)	9.92 (6.93, 13.88)	2.98 (1.92, 4.82)	− 0.70 (− 0.77, − 0.60)
Eastern sub-Saharan Africa	45,368.06 (43,021.45, 4,7641.56)	15,446.41 (14,245.45, 16,831.81)	− 0.66 (− 0.69, − 0.62)	289.82 (203.99, 404.56)	63.34 (41.17, 94.95)	− 0.78 (− 0.81, − 0.75)
Southern sub-Saharan Africa	45,687.12 (41,518.62, 49,488.72)	11,665.81 (9619.52, 14,101.60)	− 0.75 (− 0.80, − 0.69)	222.30 (135.59, 350.04)	32.75 (19.41, 53.29)	− 0.85 (− 0.89, − 0.82)
Western sub-Saharan Africa	39,074.32 (37,958.88, 40,223.94)	13,401.81 (12,561.68, 14,194.31)	− 0.66 (− 0.68, − 0.64)	261.65 (192.72, 348.82)	56.40 (38.91, 79.17)	− 0.78 (− 0.81, − 0.75)
Central sub-Saharan Africa	48,219.73 (45,048.54, 51,746.98)	22,494.63 (19,311.10, 25,719.52)	− 0.53 (− 0.61, − 0.45)	243.43 (167.67, 330.18)	65.50 (43.11, 98.66)	− 0.73 (− 0.80, − 0.62)
Australasia	0.00 (0.00, 0.00)	0.00 (0.00, 0.00)	0.00 (0.00, 0.00)	0.00 (0.00, 0.00)	0.00 (0.00, 0.00)	NA
Oceania	43,298.93 (37,749.18, 48,095.47)	19,320.15 (15,332.99, 23,817.06)	− 0.55 (− 0.65, − 0.44)	264.47 (161.94, 401.75)	93.36 (57.09, 143.72)	− 0.65 (− 0.74, − 0.52)
Andean Latin America	21,514.41 (18,587.89, 24,931.00)	30,153.70 (26,076.80, 34,143.48)	0.40 (0.13, 0.72)	53.50 (36.19, 79.86)	38.58 (23.28, 61.30)	− 0.28 (− 0.45, − 010)
Central Latin America	27,674.63 (24,903.45, 30,613.78)	15,437.95 (13,198.43, 18,381.61)	− 0.44 (− 0.54, − 0.31)	55.15 (35.64, 83.20)	18.38 (10.68, 30.09)	− 0.67 (− 0.73, − 0.59)
Southern Latin America	13,374.38 (10,055.30, 17,063.80)	6152.06 (4583.14, 8150.77)	− 0.54 (− 0.68, − 0.32)	9.38 (4.62, 17.23)	0.92 (0.49, 1.62)	− 0.90 (− 0.94, − 0.85)
Tropical Latin America	15,456.63 (11,528.80, 20,784.76)	12,453.34 (8957.63, 16,960.82)	− 0.19 (− 0.50, 0.22)	17.08 (10.99, 26.49)	12.61 (7.04, 22.10)	− 0.26 (− 0.50, 0.05)

*ASR* age-standardized rate, *DALYs* disability-adjusted life-years, *UI* uncertainty interval, *STH* soil-transmitted helminth, *NA* not applicable

STH infections were most prevalent in countries located in middle regions of Africa and Americas, and some Asian countries, with the ASPR ranging from 13,046.00 to 50,502.40 per 100,000 in 2021. While North American and European countries had an extreme low prevalence, even to zero. In some specific countries, for example, the ASPR of STH infections in Somalia was much higher than any other countries, reaching 50,502.38 (95% UI: 42,702.02, 58,117.72) per 100,000, followed by the three countries, Peru (38,256.98, 95% UI: 2085.05, 44,729.36), Nicaragua (31,918.37, 95% UI: 25,863.19, 37,674.37), Chad (30,616.25, 95% UI: 23,853.97, 38,950.70). With regard to ASR of DALYs caused by STH infections, Somalia also ranked the first with 307.46 (95 UI 187.99, 484.48) per 100,000. The ASR of DALYs in following five countries/territories, Rwanda, Chad, Guinea, Papua New Guinea, Central African Republic, ranged from 100 to 156 per 100,000.

### Trend in the globe and various SDI level

Globally, The ASPR of STH infections had an obvious decrease trend worldwide [estimate annual percent change (EAPC) = − 4.03, 95% *CI*: − 4.13, − 3.93], from 27,728.35 (95% UI:  25,660.30, 29,899.61) in 1990 to 8429.89 (95% UI: 7697.23, 9362.18) per 100,000 in 2021. During the same period, the ASPR of ascariasis dropped (EAPC = − 5.07, 95% *CI* − 5.28, − 4.85) from 15,930.11 (95% UI: 13,515.38, 18,419.92) to 3856.33 (95% UI: 3133.93, 4760.38) per 100,000; the ASPR of hookworm disease dropped (EAPC: − 5.61, 95% *CI*: − 5.95, − 5.20) from 8805.82 (95% UI: 7809.61, 10,049.50) to 1505.49 (95% UI: 1418.67, 1598.89) per 100,000; while the downward trend of trichuriasis was quite slower compared with the other two species infection (EAPC = − 2.69, 95% *CI*: − 2.91, − 2.46), from 8690.82 (95% UI: 7466.72, 10,146.42) to 3482.27 (95% UI: 3147.21, 3898.86) per 100,000. The ASR of DALYs caused by STH infections had a more pronounced decline (EAPC = − 5.71, 95% *CI*: − 5.87, − 5.55) from 103.83 (95% UI: 70.38, 153.46) to 18.84 (95% UI: 12.61, 27.31) per 100,000 during the study years, among which, the ASR of DALYs caused by ascariasis and hookworm disease dropped about sixfold, while trichuriasis dropped about fourfold.

The distribution of disease burden of STH infections was negatively correlated with SDI. In low SDI regions, the ASPR of STH infections was 15,665.55 (95% UI: 14,203.48, 17,110.50) per 100,000 in 2021, with ASR of DALYs reaching 55.06 (95% UI: 37.16, 79.09) per 100,000; while in high SDI locations, the ASPR of STH infections was 15.84 times lower than in low SDI locations [989.36 (95% UI: 774.22, 1213.66) per 100,000], and the DALYs rate was much lower [0.10 (95% UI: 0.05, 0.16) per 100,000].

**Fig. 1:**
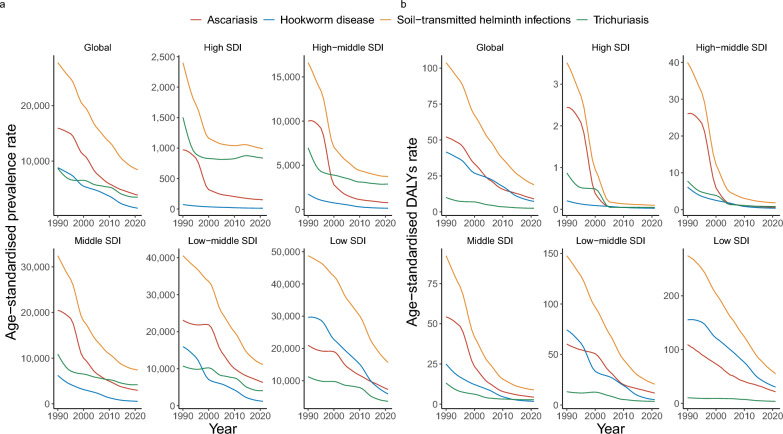
Trend of ASR of prevalence and DALYs (per 100,000) of STH infections in the globe and different SDI regions. **a** Trend of ASPR of STH infections in the globe and different SDI regions, **b** Trend of ASR of DALYs caused by STH infections in the globe and different SDI regions. *ASR* age-standardized rate, *ASPR* age-standardized prevalence rate, *DALYs* disability-adjusted life-years, *SDI* socio-demographic index, *STH* soil-transmitted helminth

The downward trend varied across different SDI regions. In high SDI regions, the ASPR of STH infections decreased (EAPC = − 2.39, 95% *CI*: − 2.94, − 1.85) from 2402.29 (95% UI: 1957.70, 2899.51) in 1990 to 1159.30 (95% UI: 913.03, 1449.96) in 2021 per 100,000, with ASR of DLAYs dropped (EAPC = − 13.16, 95% *CI*: − 14.71, − 11.57) from 3.52 (95% UI: 5.99, 1.83) to 0.95 (95% UI: 0.52, 1.63) per 100,000. So did the trend pattern in high-middle SDI regions with higher rate of prevalence and DALYs. In middle SDI regions, both the ASR of STH infections prevalence and DALYs decreased from 1990 to 2021, the former dropped (EAPC = − 5.08, 95% *CI*: − 5.30, − 4.86) from 32,436.91 (95% UI: 29,251.24, 35,967.88) to 7415.66 (6614.87, 8460.69) per 100,000, with later dropped (EAPC = − 8.18, 95% *CI*: − 8.53, − 7.83) from 92.32 (95% UI: 56.45, 143.08) to 9.09 (95% UI: 5.59, 14.43) per 100,000. Such downward pattern is similar in low-middle SDI regions. In low SDI regions, the ASPR of STH infections (per 100,000) fell (EAPC = − 3.71, 95% *CI* : − 4.05, − 3.37) from 48,748.62 (95% UI: 46,296.71, 50,880.93) to 15,665.55 (95% UI: 14,203.48, 17,110.50) with ASR of DLAYs falling (EAPC = − 5.21, 95% *CI*: − 5.52, − 4.89) from 274.59 (95% UI: 196.49, 382.67) to 55.06 (95% UI: 37.16, 79.09) during the study years. When it comes to specific species infection in 2021, the most prevalent was trichuriasis in high, high-middle and middle SDI regions, but ascariasis in low-middle and low SDI regions; While the DLAYs caused by STH infections was approaching zero in high and high middle SDI regions, but remained a serious public health problem in middle and low-middle SDI regions, DALYs caused by hookworm disease and ascariasis in low SDI regions in particular (Fig. 1).

### Age and gender differences in STH infections

**Fig. 2:**
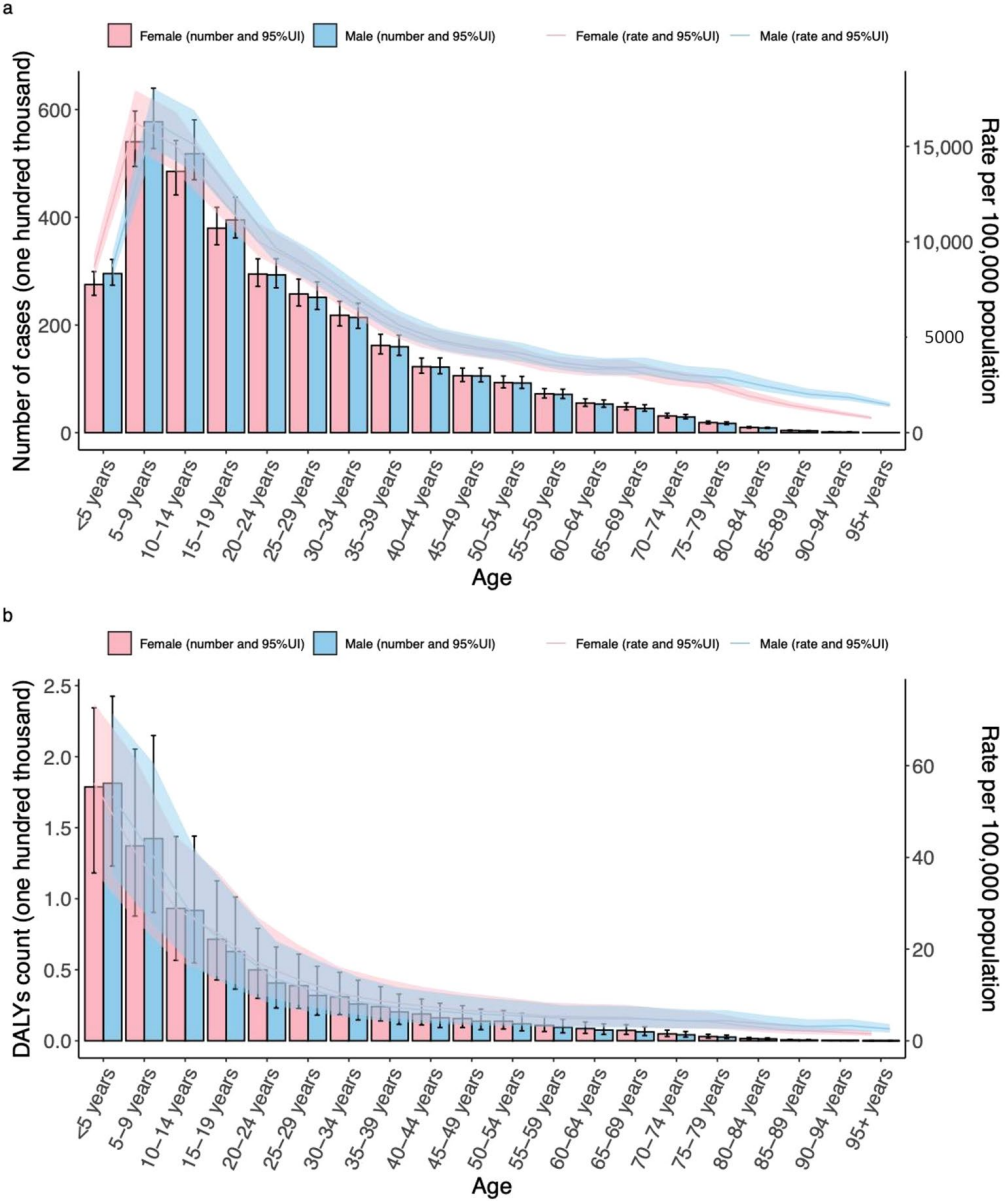
Age and gender difference in STH infections. **a** Rate of STH infections prevalence, **b** Rate of DLAYs by STH infections. *DALYs* disability-adjusted life-years, *STH* soil-transmitted helminth

There was no significant difference between genders observed in each age group. The ASR of STH infections prevalence and DALYs were various across different age intervals. The rate of prevalence was higher in the groups of 5–19 years, especially the group of 5–9 years with the rate of prevalence of 16,263 (95% UI: 14,877.06, 18,003.49) and DALYs of 40.69 (95% UI: 25.98, 60.91) per 100,000 and reduced with the increase of ages (Fig. 2). As for specific species infection, ascariasis had a similar pattern and was also most prevalent in the group of 5–14 years, with the rate of prevalence 8029.41 (95% UI: 6646.23, 9775.80) and DALYs of 13.57 (95% UI: 8.45, 21.41) per 100,000. Hookworm disease had also a highest rate in the group of 5–9 years, with the prevalence of 3687.23 (95% UI: 3462.76, 3917.55) and DALYs of 20.30 (95% UI: 12.86, 31.24) per 100,000. Trichuriasis was mainly prevalent in the group of 5–29 years, the group of 5–9 years in particular with the rate of prevalence of 5578.97 (95% UI: 5092.24, 6159.89) and DALYs of 20.30 (95% UI: 12.86, 31.24) per 100,000 (age and gender difference for specific species infections, Supplementary Figure 1).

### Association between STH infections and SDI in 21 geographical regions

**Fig. 3:**
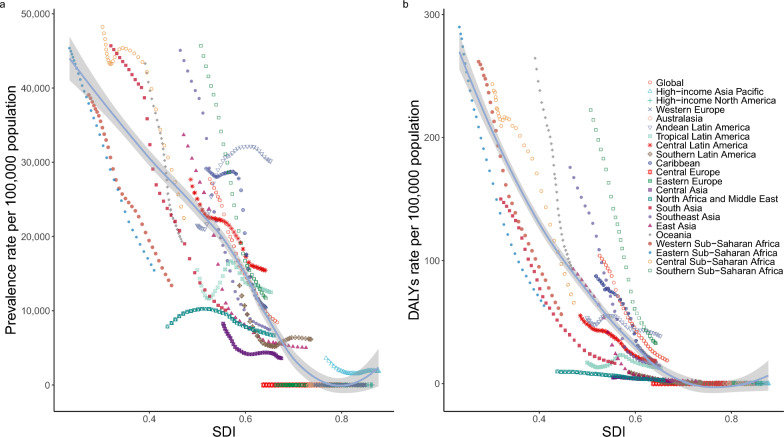
ASR of prevalence (**a**) and DALYs (**b**) of STH infections in 21 GBD regions by SDI, 1990–2019. Expected values, based on SDI and disease rates in all locations, are shown as a solid line; expected values based on a calculation accounting for the SDI and disease rates across all locations. 30 points are plotted for each region and show the observed age-standardized rate of prevalence or DALYs for each year from 1990 to 2021 for that region. The shaded area indicates the 95% confidence interval of the expected values. Points above the solid line represent a higher-than-expected burden, and those below the line show a lower-than expected burden. *ASR* age-standardized rate, *DALYs* disability-adjusted life-years, *GBD* Global Burden of Diseases, *SDI* socio-demographic index, *STH* soil-transmitted helminth

At regional level, the ASRs of STH infections prevalence (*r* = − 0.8807, *P* < 0.0001) and DALYs (*r* = − 0.9069, *P* < 0.0001) were negatively correlated with SDI among regions. Southern sub-Saharan Africa, Latin America and Caribbean had higher-than-expected ASPR, while central Asia and central Europe had a lower-than-expected ASPR based on their SDI between 1990 and 2021 (Fig. 3).

The ASRs of ascariasis prevalence (*r* = − 0.8561, *P* < 0.0001) and DALYs (*r* = − 0.9064, *P* < 0.0001) were negatively correlated with SDI among regions. The ASR of ascariasis was scattered higher and lower of the expected value between 1990 and 2021. Oceania had a lower-than-expected ASPR based on their SDI.

The ASRs of hookworm disease prevalence (*r* = − 0.8826, *P* < 0.0001) and DALYs (*r* = − 0.8840, *P* < 0.0001) were negatively correlated with SDI among regions. Southern sub-Saharan Africa had higher-than-expected ASPR, while western sub-Saharan Africa, South Asia, and North African and Middle East had a lower-than-expected ASPR based on their SDI between 1990 and 2021.

The ASRs of trichuriasis prevalence (*r* = − 0.7164, *P* < 0.0001) and DALYs (*r* = − 0.7841, *P* < 0.0001) were also negatively correlated with SDI among regions. Central Latin America had higher-than-expected ASPR, while North African and Middle East and Central Asia had a lower-than-expected ASPR based on their SDI between 1990 and 2021 (Supplementary Figure 2).

## Discussion

This study confirms the previous estimation of the reduction of the global burden due to STH infections [6]. The decline may be largely attributable to the improvement of social economic level [14, 15] and several core strategic interventions. For instance, preventive chemotherapy for preschool and school-aged children, and women of child-bearing age [16, 17]; access to adequate sanitation and waste management facilities, improved hygiene practices, and safe water at the household level and in schools; improved diagnostics and treatment to individuals living in STH endemic areas [18, 19]; and health education to high risk groups for behavior change to reduce the probability of transmission [20].

The distribution of STH infections varies across different SDI level and geographical regions. We found that the sub-Saharan African regions, middle part of Americas where with lower SDI remain the region with the larger STH burden in terms of prevalence and DALYs rates. These findings coincide with the risk mapping study of STH infection [7, 8]. A combination of factors, poverty, socioeconomic status, access to sanitation facility, and long-lasting defecation behavior could be the main drivers of the distribution of STH infections. In addition, different natural factors, including the higher temperature, rainfall, normalized difference vegetation index, in different regions might accelerate the differentiated distribution of STH infections [10].

The number of DALYs due to STH infection was estimated to be at around 5.99 million in 1990, 4.22 million in 2000, dropping to 2.49 million in 2010 and to an estimated 1.45 million in 2020. When observing the contribution of specific parasite species and its control intervention, the reduction in absolute DALYs has been more attributable to the falling in hookworm disease (from 2.37 million DALYs in 1990 to 0.57 million DALYs in 2020), while the decrease in DALYs has been more pronounced for trichuriasis (from 0.58 million DALYs in 1990 to 0.19 million DALYs in 2021). The reduction seems more obvious in school-aged and preschool-aged children, but that the decline has also been observed in other age groups [6]. The long-term preventive chemotherapy intervention may contribute largely to the reduction of DALYs [21, 22], particularly in the children group who are recognized as the more active in terms of open defecation [23] which exacerbates the transmission of STH infections.

Although the number of school-age children treated with preventive chemotherapy has progressed steadily from less than 120 million in 2008 to over 450 million in 2018 and achieved the reduction of STH-attributable prevalence in preschool-and school-age children [4, 16], the prevalence and DALYs of STH infections in school-age children still higher than other groups, which is consistent with WHO’s identification and results of many studies [24]. The countries that started preventative chemotherapy programs early have already reached a low prevalence level of STH infections. If the implementation of preventive chemotherapy continues to scale up, it is estimated that most STH-endemic countries will eliminate STH infections among preschool- and school-age children by 2025 [25].

In order to accelerate the elimination of STH infections as a public health problem, WHO representatives, experts from STH-endemic countries, and relative partners recommend six targets to eliminate STH for 2030 in Basel, Switzerland in 2018 [26]. Highlighted issues were identified as follows: (i) reduce of STH morbidity in preschool- and school-age children; (ii) reduce the reliability of preventive chemotherapy; (iii) call for more domestic financial support for STH control; (iv) scale up the control programs among specific populations, such as adolescent, pregnant and women of reproductive age; (v) include an efficient strongyloidiasis control program in STH control; and (vi) enhance access to basic sanitation and hygiene facility in STH-endemic areas. However, 5 years later, the huge gap between challenge and achievement still remained and actions required [27]: increase political commitment for sustainable domestic financing; mitigate the potential drug resistance; enhance surveillance and mapping to monitor the progress and for target treatment [28]; scale up and improve survey to evaluate the impact of integrated intervention, economic level and preventive chemotherapy; formulate and implement One Health approach [29] for the elimination STH infection as a public health problem [30, 31].

This study analyzed the ASR of prevalence and DALYs caused by STH infections based on data of GBD 2021 from the perspective of decline trend, geographical distribution in 21 regions and 204 countries and territories, relationship with age intervals and SDI, which provide a comprehensive and opportune oversight of STH infections disease burden worldwide. However, the estimated burden data cannot replace and/or the high-quality primary data. Trend, distribution, and correlations of STH infections assessed in this study should be interpreted with caution because of the following reasons: the estimation of the burden depends on the availability and quality of the primary data in GBD 2021 which may encounter the problem of missing raw data in some countries, lower quality of data and collection methods, and inconsistencies of primary epidemiological data and different diagnosis and detection methods which might affect the comparability of results [11]. While our study provides valuable insights into the socio-demographic factors associated with parasitic infections across different geographic regions, it is important to acknowledge certain limitations due to the lack of environmental data. Environmental factors such as temperature, humidity, soil pH, and rainfall patterns significantly influence the development and transmission of parasites. This absence of detailed environmental data limits our ability to fully account for these influential factors, which may result in an incomplete understanding of the dynamics affecting parasitic prevalence. To address this limitation, future studies should strive to include comprehensive environmental data to better elucidate the interplay between socio-demographic and environmental factors in parasitic infections. Besides, there was an absence of age and gender stratified comparison analysis in different SDI and geographical regions. Furthermore, strongyloidiasis is an intestinal helminth which infected more than 600 million globally, while enterobiasis is a parasitic worm infection usually reported in the United States and Western Europe, and their burden should be quantified precisely [32]. The two infections are usually being discussed within the category of STH infections but not in this study [2, 33, 34]. To address above limitations, we advocate for the development of diverse analytical methods that validate the results of this study and extend the disease scope for further study.

## Conclusions

STH infections are still a public health problem worldwide, particularly in the middle regions of Africa and Americas. Compare with other ages groups, populations at 5–19 age intervals are of higher prevalence and DALYs. Reduction of the ASPR and the loss of DALYs are negatively correlate with the increase of SDI. We may consider increasing political commitment to ensure sustainable financing support, develop more accurate surveillance and mapping systems for target control, monitor drug resistance and develop more effective diagnostics and medicine, and conduct the One Health approach to accelerate the progress of STH infections elimination.

## Supplementary Information

Supplementary Table 1. Global burden of soil-transmitted helminth infections.

Supplementary Figure 1. Age and gender difference in STH infections and specific species infections. a. ASPR of ascariasis, b. ASPR of hookworm disease, c. ASPR of trichuriasis; d. ASR of ascariasis DALYs lost, e. ASR of hookworm disease DALYs lost, f. ASR of trichuriasis DALYs lost. ASPR: age-standardized prevalence rate, ASR: age-standardized rate, DALYs: disability-adjusted life-years, STH infections: soil-transmitted helminth-related infectious diseases of poverty. Supplementary Figure 2. ASPR of ascariasis, hookworm disease, and trichuriasis in 21 GBD regions by SDI, 1990–2021. ASPR: age-standardized prevalence rate, GBD: Global Burden of Diseases, SDI: socio-demographic index. 

## Data Availability

The data obtained from the Global Burden of Diseases, Injuries, and Risk Factors Study (GBD) 2021 were open access.

## Abbreviations

ASR: Age-standardized rate
ASPR: Age-standardized prevalence
*CI*: Confidence interval
DALYs: Disability-adjusted life-years
EAPC: Estimate annual percent change
GBD: Global Burden of Diseases
SDI: Socio-demographic index
STH: Soil-transmitted helminth
UI: Uncertainty interval
WHO: World Health Organization
