# Supplementary figures and images for: Global burden of soil-transmitted helminth infections, 1990–2021
Source: Infect Dis Poverty. 2024 Oct 24;13:77. doi: 10.1186/s40249-024-01238-9 (PMC11515461; doi:10.1186/s40249-024-01238-9)

## Slide 1
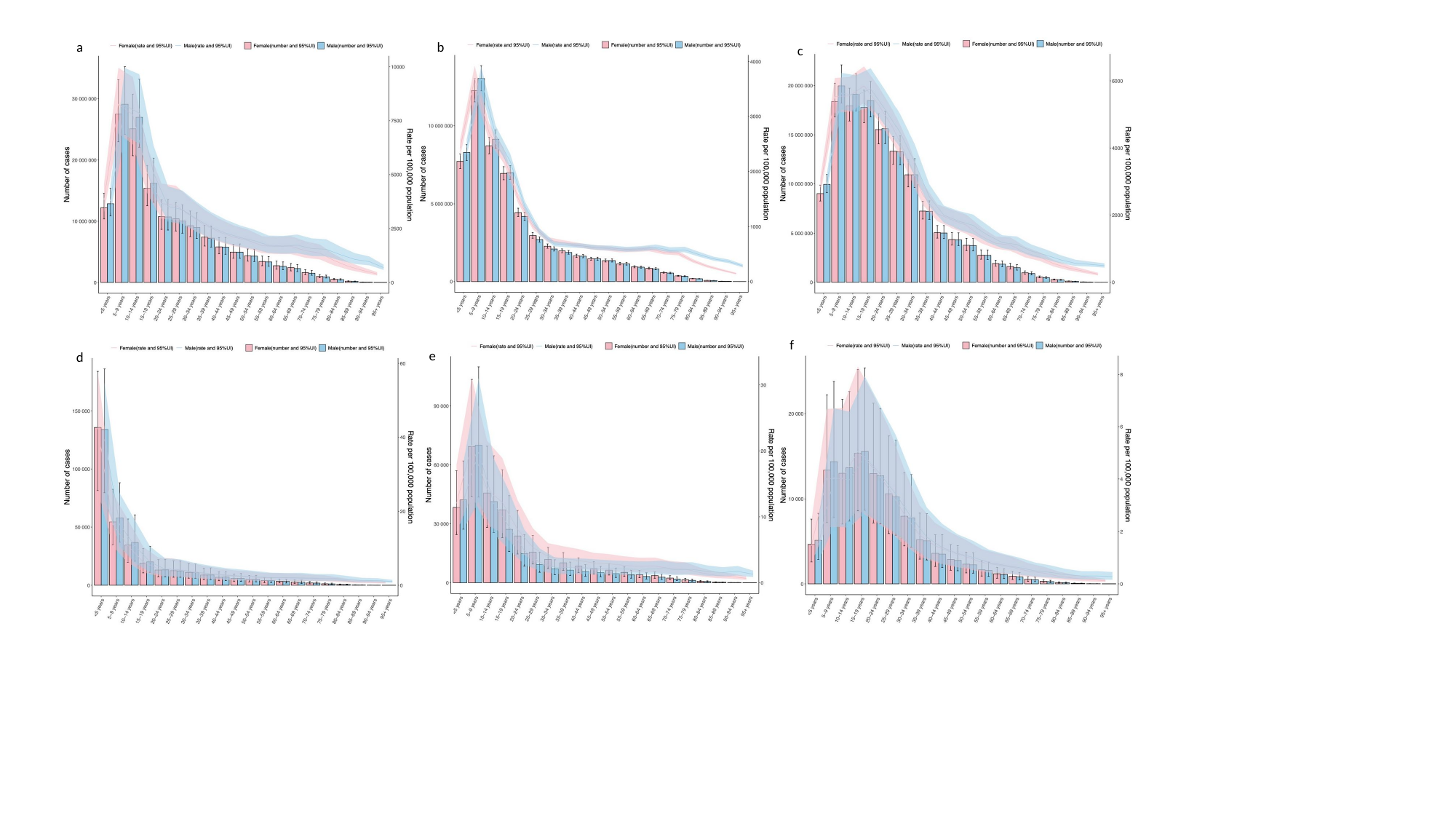

a
b
c
f
e
d

## Slide 2
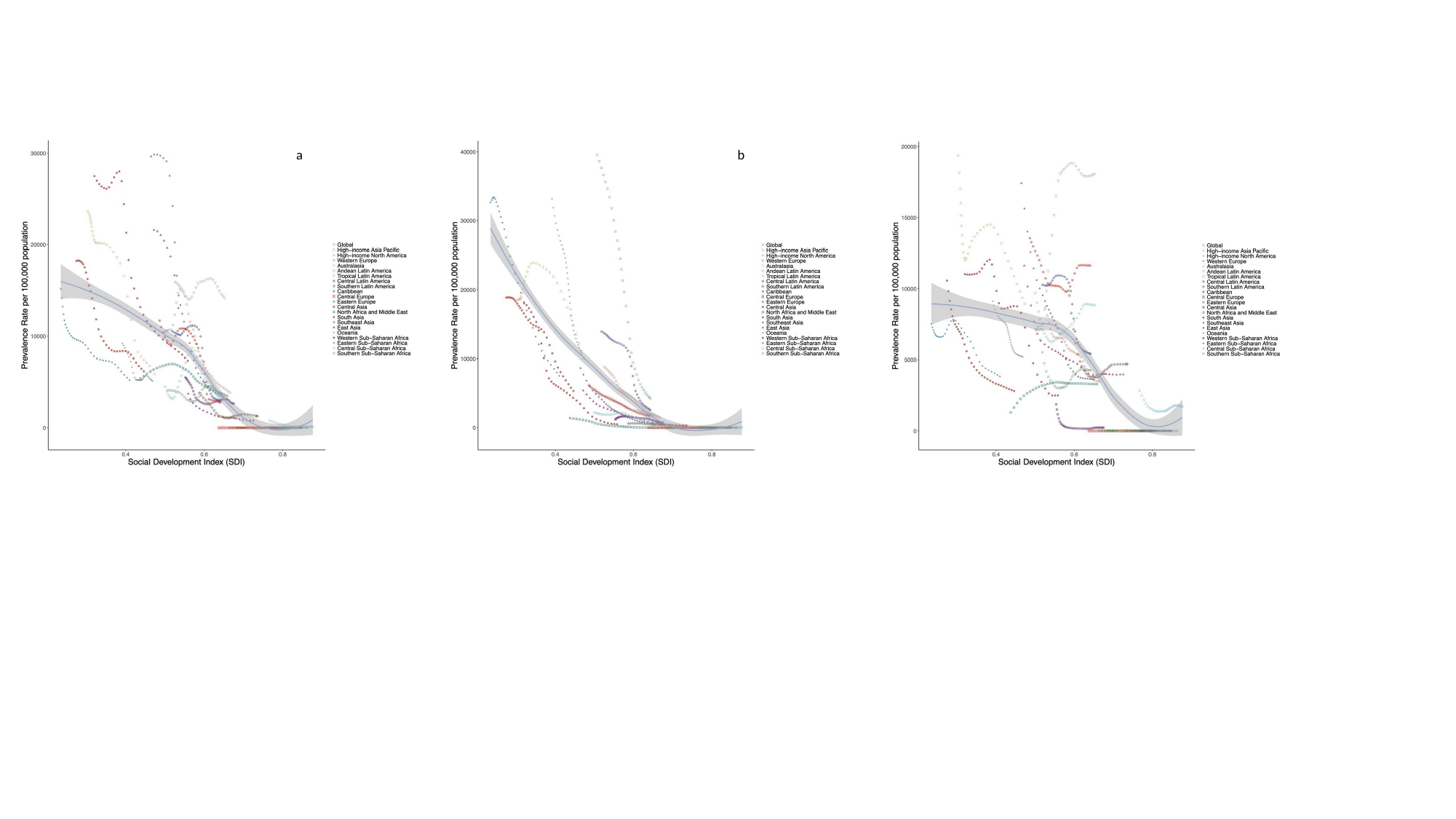

a
b

Supplement: Supplementary file 2 — Supplementary Figure 1. Age and gender difference in STH infections and specific species infections. a. ASPR of ascariasis, b. ASPR of hookworm disease, c. ASPR of trichuriasis; d. ASR of ascariasis DALYs lost, e. ASR of hookworm disease DALYs lost, f. ASR of trichuriasis DALYs lost. ASPR: age-standardized prevalence rate, ASR: age-standardized rate, DALYs: disability-adjusted life-years, STH infections: soil-transmitted helminth-related infectious diseases of poverty. Supplementary Figure 2. ASPR of ascariasis, hookworm disease, and trichuriasis in 21 GBD regions by SDI, 1990–2021. ASPR: age-standardized prevalence rate, GBD: Global Burden of Diseases, SDI: socio-demographic index. [file 40249_2024_1238_MOESM2_ESM.pptx]
